# Good long-term visual outcomes of parapapillary choroidal melanoma patients treated with proton therapy: a comparative study

**DOI:** 10.1007/s10792-020-01594-z

**Published:** 2020-09-25

**Authors:** Alessia Pica, Damien C. Weber, Laureen Vallat, Ciara Bergin, Jan Hrbacek, Claude Schweizer, Leonidas Zografos, Ann Schalenbourg

**Affiliations:** 1 Center for Proton Therapy, Paul Scherrer Institute, ETH Domain, 5232 Villigen, Switzerland; 2 Department of Ophthalmology, University of Lausanne, Jules-Gonin Eye Hospital, Fondation Asile des Aveugles, Lausanne, Switzerland

**Keywords:** Uveal melanoma, Proton radiation therapy, Optic nerve head, Visual acuity

## Abstract

**Purpose:**

To evaluate why small- and certain medium-sized parapapillary choroidal melanoma (pcM) patients treated with hypo-fractionated proton therapy (PT) retain excellent long-term visual acuity (VA) and assess the negative predictive factors for retaining good vision (≤ 0.2 logMAR (≥ 0.6 decimal) after 5 years.

**Methods:**

This single-center, retrospective, comparative study recruited consecutive pcM patients that were treated with PT. Between 1984 and 2005, 609 patients received a total of 60 CGE, of whom 310 met the following inclusion criteria: posterior tumor border ≤ 2.5 mm from the optic disc, largest tumor diameter ≤ 17.9 mm, tumor thickness ≤ 5.2 mm and available follow-up data for at least 5 years.

**Results:**

Mean follow-up was 120.8 ± 48.8 months (54.0–295.0). Out of 310 patients, 64 (21%) maintained a VA ≤ 0.2 logMAR (≥ 0.6 decimal) for at least 5 years following PT and were allocated to the “good visual outcome” (GVO) group, while the remaining 246 (79%) constituted the “poor visual outcome” (PVO) group, subdivided into 70 (22%) with a VA of 0.3–1.0 logMAR (0.1–0.5 decimal) and 157 (57%) patients with a VA > 1.0 logMAR (< 0.1 decimal). On multivariate analysis, older age (*P* = 0.04), tumor localization ≤ 0.5 mm to the fovea (*P* < 0.03), volume of the optic disc and macula receiving 50% of dose (30 CGE) (*P* = 0.02 and *P* < 0.001, respectively) were independent negative predictors of GVO.

**Conclusions:**

Of 310 small- to medium-sized pcM patients successfully treated with PT, 21% retained a VA ≤ 0.2 logMAR (≥ 0.6 decimal) for at least 5 years. Strongest negative predictive factor for retaining good long-term vision was the volume of the macula irradiated with at least 30 Gy.

## Introduction

Over the last decades, conservative radiation therapy has gradually replaced enucleation as the preferred treatment modality for small- to medium-sized choroidal melanoma. In 2006, the COMS-study confirmed that the 12-year metastatic death risk was identical for either treatment type [1]. Parapapillary choroidal melanoma (pcM) is often treated with external beam proton therapy (PT), rather than brachytherapy, to decrease the risk of a local recurrence through geographical miss. However, the indication for conservative PT of pcM is still challenged, because irradiation of the optic disc is correlated with a higher risk of complications as a result of direct neuropathic effects and a radiation-induced vasculopathy, leading to loss of useful vision and even secondary enucleation [2, 3].

Since 2011, three research groups [4–6] have published results on PT outcomes specifically in pcM. Lane et al. [4] reported a melanoma-related mortality rate of 24% at 15 years, and a local recurrence rate of 3.3 and 6% at 5 and 10 years, respectively. Secondary enucleation rates varied from 13.3 to 9.5% at 5 years and 17.1 to 10.7% at 10 years according to Lane [4] and Riechardt et al. [5], respectively.

Surprisingly, they found that 20.3% [4] and 14% [5] of these challenging patients kept a useful vision of at least 1.0 logMAR (0.1 decimal) after five years in the treated eye following hypo-fractionated PT, despite a high-radiation dose delivered to the optic disc, and Thariat et al. [6] reported that 31.9% had only a relative visual acuity (VA) loss of ≤ 0.3 logMAR between 2 and 5 years following PT. Why did certain eyes with pcM keep perfect vision up to 5 years following PT, while others with apparently similar tumors lost perception of light completely? What were the most important predictive factors for long-term vision? To answer these questions, we took a different approach and designed a comparative study, analyzing which parameters in patients with excellent long-term vision (≤ 0.2 logMAR (≥ 0.6 decimal) differed mostly from other patients.

## Methods

This single-center, retrospective, interventional, comparative study was approved by the ethical committee of the Canton of Vaud, Switzerland (authorization #2016–01,861) and complies with the Declaration of Helsinki.

Between 1984 and 2005, we identified 609 patients treated with PT for a pcM, i.e., with a posterior tumor border located within a radius of 2.5 mm from the optic disc. We defined a largest tumor diameter (LTD) of ≤ 17.9 mm, the upper limit of medium-sized (T3) tumors according to the American Joint Committee on Cancer (AJCC) [7]. Maximum tumor thickness was defined by the height of the thickest pcM case that still managed to maintain a VA of ≤ 0.2 logMAR (≥ 0.6 decimal); this height was 5.2 mm and thus, the upper limit of tumor thickness for inclusion in our study was 5.2 mm. From this sample of 609 patients, we excluded 299 patients. For most of those (*n* = 295), we did not have follow-up data for at least 5 years following PT, including 10 patients who underwent secondary enucleation because of radiation-induced complications (*n* = 8) or because of a local recurrence (*n* = 2). Four more patients presenting a local recurrence were excluded because they underwent a second radiation therapy. The remaining 310 patients were included in our analyses. In 30 of these cases, presenting initially with a clinical differential diagnosis of suspicious nevus versus small choroidal melanoma, a periodic observation had been performed in order to document proof of growth before PT.

A best corrected visual acuity (BCVA) of ≤ 0.2 logMAR (≥ 0.6 decimal) for at least 5 years following PT was considered as a ‘good long-term’ VA. Our study compared patient, ocular and tumor characteristics at baseline (pre-treatment), PT parameters, as well as follow-up BCVA and ocular complications of patients with a good long-term VA, allocated to the “good visual outcome” (GVO) group, with those of the remaining patients, representing the “poor visual outcome” (PVO) group. The latter comprises both ‘medium visual outcome’ (MVO) patients, with still legal vision, i.e., a BCVA of 0.3–1.0 logMAR (0.1–0.5 decimal) and ‘low visual outcome’ (LVO) patients, legally blind in the treated eye with a BCVA > 1.0 logMAR (< 0.1 decimal).

PT was planned and delivered as described previously [8–10]. In brief, the tumor location and extension were determined during tantalum clip surgery under general anesthesia at the Jules-Gonin Eye Hospital (HOJG, Lausanne, Switzerland). PT was planned and delivered at the Paul Scherrer Institute (PSI, Villigen, Switzerland), using the EYEPLAN software program and a custom-modeled head holder for immobilization [10]. A total dose of 54.5 Gy, corresponding to 60 CGE (Cobalt Gray Equivalent), was delivered in 4 fractions of 15 CGE on 4 consecutive days. Safety margins were 2.0 mm for the lateral and 2.5 mm for the distal and proximal margins. All fields were defined by the 90% isodose level. The chosen gazing angle resulted from the best perceived compromise between the automatically EYEPLAN generated percentages of irradiation to critical structures (optic disc, fovea, lens, etc.) for each proposed fixation as well as the expected long-term radiation side effects specific to each of those structures and the patient’s capacity of maintaining that fixation for the duration of the treatment.

Following treatment, patients were asked to return to the HOJG for follow-up examinations at 6 months, 1.5 years, 3 years, 5 years and every 2.5 years thereafter, or as clinically indicated. Standard examination at each follow-up visit included BCVA, slit-lamp examination, intra-ocular pressure measurement, dilated indirect ophthalmoscopy, along with color fundus photography and B-scan ultrasonography to assess local tumor control. When patients were unable to return to Lausanne (mainly those living more than 500 km away), follow-up data were collected by contacting the referring ophthalmologist.

The data systematically collected included patient characteristics such as age and general health data (diabetes mellitus, arterial hypertension, anticoagulant therapy), ophthalmic parameters including BCVA before PT (baseline), at 5-years post-treatment (follow-up) and at the last follow-up examination, presence of a foveal detachment before PT and the eye’s axial length. Baseline tumor characteristics included tumor size (LTD, thickness), and location of the posterior tumor border with regard to the optic disc (abutting or not), and with regard to the fovea (temporal vs. nasal; distance from the fovea ≤ 0.5 mm, between 0.6 and 2.5 mm, > 2.5 mm). Recorded PT parameters were the dose-volume histograms of the optic disc, optic nerve and macula. In EYEPLAN, critical structures like the optic nerve, the optic disc and the macula are positioned within the ellipsoid homogeneous EYEPLAN eye model, a three-dimensional (3D) reconstruction of the eye, based on pre- and peroperative measures (e.g., axial length, limbus diameter…), and not on 3D-imaging. In consequence, the dose-volume histogram evaluation is also based on this model, the dose levels being reported as 90, 50 and 20% of the total dose, i.e., 60 CGE. Follow-up data other than BCVA included complications such as radiation-induced optic neuropathy and maculopathy as well as non-radiation-induced (‘other’) maculopathies, neovascular glaucoma and optic atrophy.

Before statistical analyses, all BCVA data were converted into logMAR using the logMAR = [−log (decimal)] formula [11]. When BCVA corresponded to “counting fingers”, “hand movements”, or “light perception”, the Freiburg Visual Acuity Test (FrACT) was used to define a value [12].

Statistical analyses were performed using the R software package [13]. The GVO and PVO groups were compared using the log-rank test statistic at 5% level of significance employing the “survival” R package [https://cran.r-project.org/package=survival]. Data were censored at the last available follow-up visit or at the moment of enucleation/local recurrence. The predictive factors for visual impairment were examined using univariate logistic regression. Parameters reaching statistical significance with univariate logistic regression (*P* < 0.05) were included in a subsequent multivariate analysis. The probability of retaining good long-term vision with respect to tumor location was analyzed with the Kaplan–Meier method.

## Results

**Fig. 1:**
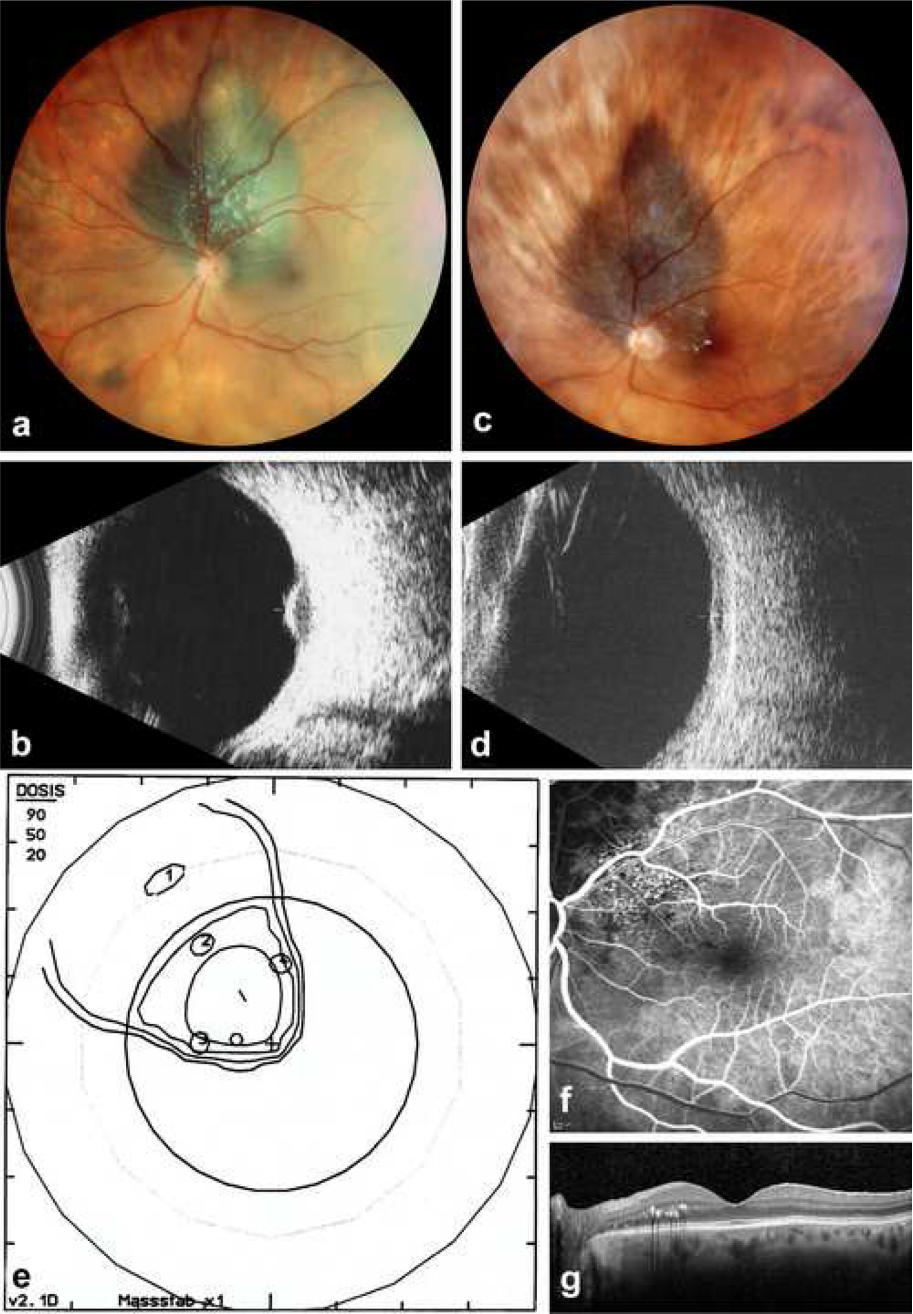
Parapapillary choroidal melanoma (left eye) maintaining useful vision following proton therapy. **a** Panoramic fundus photo (Panoret camera) in a 71-year-old female at initial presentation with loss of VA to 0.6 decimal related to a secondary macular detachment. **b** On B-scan ultrasonography (10 MHz), thickness is 3.4 mm. **c** Eleven years after radiation therapy, the tumor borders are under control on panoramic fundus photography, with some lipid exudates close to the macular border. **d** On B-scan ultrasonography (10 MHz), the atrophic scar has a residual thickness of 1.3 mm. **e** Proton therapy irradiation plan—fundus view—illustrating the tumor base, localized by four tantalum clips and surrounded by the 90, 50 and 20% isodose borders. **f** Fluorescein angiography (early venous phase) of the macula, on which radiation-induced extrafoveal telangiectasia can be identified, explaining the lipid exudates. **g** On B-scan OCT, the fovea appears normal, with some extrafoveal lipid exudates. VA is 1.0 decimal

Out of 310 pcM patients who had received PT, 64 patients (21%) maintained a VA of ≤ 0.2 logMAR (≥ 0.6 decimal) for at least 5 years following treatment and were therefore allocated to the GVO group (Fig. 1), while the remaining 246 patients (79%) entered the PVO group. In the latter, two patients presented a recurrence at 8 and 9 years following PT and were treated with a second PT, followed in the latter case by a secondary enucleation 3 years later.

Table 1 provides an overview of patient, baseline ocular and tumor characteristics, as well as ocular follow-up data, comparing patients who maintained 5 years after PT a good vision (‘GVO’ ≤ 0.2 logMAR (≥ 0.6 decimal), with those with poor vision (’PVO’ > 0.2 logMAR (≤ 0.5 decimal), subdivided in this table into those presenting medium, but still legal vision (‘MVO’ = 0.3–1.0 logMAR (= 0.1–0.5 decimal) and those with low vision, i.e., legally blind in the treated eye (‘LVO’ > 1.0 logMAR (< 0.1 decimal). Table 2 illustrates which of those parameters significantly differed between the GVO group and the PVO group (MVO + LVO patients combined). Tables 3 and 4 compare the PT parameters, deducted from the optic disc, optic nerve and macula dose-volume histograms, between the GVO and PVO groups. Based on those data, a univariate and multivariate logistic regression analysis was performed to identify the prognostic long-term VA factors, and the results are detailed in Table 5.

**Table 1:** Overview of patient, baseline ocular and tumor characteristics (before PT), as well as ocular follow-up data, comparing patients who maintained five years after PT a good vision (*‘GVO’* ≤ 0.2 logMAR (≥ 0.6 decimal), with those with poor vision (*’PVO*’ > 0.2 logMAR (≤ 0.5 decimal), subdivided in this table into those presenting medium, but still legal vision (*‘MVO’* = 0.3–1.0 logMAR (= 0.1–0.5 decimal) and those with low vision, i.e., legally blind in the treated eye (*‘LVO’* > 1.0 logMAR (< 0.1 decimal)

	GVO	PVO		Total
		MVO	LVO	
**Patient characteristics**				
No. of eyes	64 (21%)	70 (22%)	176 (57%)	310 (100%)
Age at PT (years)				
Mean	49.2 ± 12.8	51.7 ± 12.2	55.3 ± 13.5	53.2 ± 13.2
Range	20.3–74.3	24.4–80.0	15.4–83.8	15.4–83.8
General health: No. of patients with				
Diabetes	4 (6%)	1 (1%)	11 (6%)	16 (5%)
Arterial hypertension	11 (17%)	8 (11%)	40 (23%)	59 (19%)
Anticoagulant therapy	4 (6%)	0	9 (5%)	13 (4%)
**Baseline ocular characteristics**				
Visual acuity (logMAR)				
Mean	0.1 ± 0.2	0.3 ± 0.4	0.5 ± 0.6	0.4 ± 0.5
Median	0.0	0.2	0.3	0.2
Range	− 0.2 to 0.7	− 0.2 to 2.0	− 0.2 to 2.3	− 0.2 to 2.3
Foveal detachment before PT	20 (31%)	42 (60%)	91 (52%)	152 (49%)
Mean axial length (mm)	24.4 ± 3.3	24.5 ± 0.9	24.9 ± 1.1	24.7 ± 1.8
**Baseline tumor characteristics**				
Tumor size				
Median LTD (range) (mm)	11.6 (5.0–17.9)	11.6 (6.0–17.5)	12.2 (5.0–17.9)	12.0 (5.0–17.9)
Median maximal tumor height (range) (mm)	3.0 (1.5–5.2)	3.4 (1.6–5.2)	3.5 (1.5–5.2)	3.4 (1.5–5.2)
Location of posterior tumor border				
Abutting optic disc	22 (34%)	29 (41%)	98 (56%)	149 (48%)
With respect to fovea				
Temporally	16 (25%)	30 (43%)	90 (51%)	136 (44%)
Distance, i.e.,				
≤ 0.5 mm	8 (12%)	38 (54%)	129 (73%)	175 (56%)
0.6–2.5 mm	23 (36%)	21 (30%)	33 (19%)	77 (25%)
> 2.5 mm	33 (52%)	11 (16%)	14 (8%)	58 (19%)
**Ocular follow-up data**				
Radiation-induced optic neuropathy	18 (28%)	23 (33%)	92 (52%)	133 (43%)
Radiation-induced maculopathy	15 (23%)	43 (61%)	122 (69%)	180 (58%)
Other maculopathy	15 (23%)	26 (37%)	44 (25%)	85 (27%)
Neovascular glaucoma	0	2 (3%)	9 (5%)	11 (4%)
Optic atrophy	18 (28%)	39 (56%)	115 (65%)	172 (55%)

*GVO* good visual outcome*, LTD* largest tumor diameter*, LVO* low visual outcome*, MVO* medium visual outcome*, PT* proton therapy*, PVO* pour visual outcome

**Table 2:** Statistical analysis of patient, baseline ocular and tumor characteristics, as well as ocular follow-up data, comparing patients who still maintained five years after PT a good vision (*‘GVO’* ≤ 0.2 logMAR (≥ 0.6 decimal), with those who did not, i.e., both those presenting medium as well as low vision (*‘MVO’* + *‘LVO’* = *’PVO*’ > 0.2 logMAR (≤ 0.5 decimal) (total No. of patients = 310)

	GVO	PVO (MVO + LVO)	Total	*P* value
**Patient characteristics**				
No. of eyes	64 (21%)	246 (79%)	310 (100%)	
Follow-up (months)				
Mean	131.3 ± 49.3	118.1 ± 48.5	120.8 ± 48.8	0.053
Median	124.5	109.0	115.0	0.02
Range	61.0–280.0	54.0–295.0	54.0–295.0	
Age at PT (years)				
Mean	49.2 ± 12.8	54.3 ± 13.1	53.2 ± 13.2	**0.005**
Range	20.3–74.3	15.4–83.8	15.4–83.8	
General health: No. of patients with				
Diabetes	4 (6%)	12 (5%)	16 (5%)	0.68
Arterial hypertension	11 (17%)	48 (20%)	59 (19%)	0.62
Anticoagulant therapy	4 (6%)	9 (4%)	13 (4%)	0.35
**Baseline ocular characteristics**				
Visual acuity (logMAR)				
Mean	0.1 ± 0.2	0.5 ± 0.6	0.4 ± 0.5	** < 0.001**
Median	0.0	0.3	0.2	** < 0.001**
Range	− 0.2 to 0.7	− 0.2 to 2.3	− 0.2 to 2.3	
Foveal detachment before PT	20 (31%)	132 (54%)	152 (49%)	**0.001**
Mean axial length (mm)	24.4 ± 3.3	24.8 ± 1.0	24.7 ± 1.8	0.06
**Baseline tumor characteristics**				
Tumor size				
Median LTD (range) (mm)	11.6 (5.0–17.9)	12.0 (5.0–17.9)	12.0 (5.0–17.9)	0.067
Median maximal tumor height (range) (mm)	3.0 (1.5–5.2)	3.4 (1.5–5.2)	3.4 (1.5–5.2)	**0.004**
Location of posterior tumor border				
Abutting optic disc	22 (34%)	127 (52%)	149 (48%)	** < 0.001**
With respect to fovea				
Temporally	16 (25%)	120 (49%)	136 (44%)	**< 0.001**
Distance, i.e.,				**< 0.001**
≤ 0.5 mm	8 (12%)	167 (68%)	175 (56%)	
0.6 – 2.5 mm	23 (36%)	54 (22%)	77 (25%)	
> 2.5 mm	33 (52%)	25 (10%)	58 (19%)	
**Ocular follow-up data**				
Radiation-induced optic neuropathy	18 (28%)	115 (47%)	133 (43%)	**0.007**
Radiation-induced maculopathy	15 (23%)	165 (67%)	180 (58%)	**< 0.001**
Other maculopathy	15 (23%)	70 (28%)	85 (27%)	0.74
Neovascular glaucoma	0	11 (4%)	11 (4%)	0.20
Optic atrophy	18 (28%)	154 (63%)	172 (55%)	** < 0.001**

*GVO* good visual outcome*, LTD* largest tumor diameter*, LVO* poor visual outcome*, MVO* medium visual outcome*, PT* proton therapy*, PVO* poor visual outcome

**Table 3:** Comparative analysis between the GVO and PVO groups of the PT parameters related to the position of the posterior tumor border with regard to the OD: statistical distribution within each group of the number of cases touching or not touching the OD and the ensuing mean % of OD surface and mean ON length that was irradiated with 20%, 50% and 90% of the total 60 CGE dose

PT parameters	GVO			PVO		
Position of the posterior tumor border relative to the OD	Touching	Not touching	Total	Touching	Not touching	Total
No. of eyes (% of the group)	22 (34%)	42 (66%)	64 (100%)	127 (52%)	119 (48%)	246 (100%)
Mean % of OD surface irradiated at						
20% of dose (15 CGE)	100	63	76	100	74	88
50% of dose (30 CGE)	100	52	69	100	62	82
90% of dose (50 CGE)	100	34	57	99	43	73
Mean length (mm) of ON irradiated at						
20% of dose (15 CGE)	4.2	1.9	2.6	4.6	2.3	3.5
50% of dose (30 CGE)	3.8	1.3	2.2	4.2	1.7	3.0
90% of dose (50 CGE)	3.3	0.8	1.6	3.6	1.0	2.3

*CGE* Cobalt Gray Equivalent*, GVO* good visual outcome*, OD* optic disc*, ON* optic nerve*, PT* proton therapy*, PVO* poor visual outcome

**Table 4:** Comparative analysis between the GVO and PVO groups of the PT parameters related to the position of the posterior tumor border with regard to the fovea: statistical distribution within each group of the number of cases with tumors located at ≤ 0.5, between 0.6 and 2.5, or > 2.5 mm from the fovea and the ensuing mean % of macular surface that was irradiated with 20%, 50% and 90% of the total 60 CGE dose

PT parameters	GVO				PVO			
Distance between the posterior tumor border and the fovea (mm)	≤ 0.5	0.6–2.5	> 2.5	Total	≤ 0.5	0.6–2.5	> 2.5	Total
No. of eyes (% of the group)	8 (12%)	23 (36%)	33 (52%)	64 (100%)	167 (68%)	54 (22%)	25 (10%)	246 (100%)
Mean % of macular surface irradiated at								
20% of dose (15 CGE)	100	91	10	50	100	97	41	93
50% of dose (30 CGE)	100	74	4	42	100	90	23	89
90% of dose (50 CGE)	100	53	0	31	100	78	8	85

*CGE* Cobalt Gray Equivalent*, GVO* good visual outcome*, PT* proton therapy*, PVO* poor visual outcome

**Table 5:** Logit Regression Analysis, identifying negative predictive factors for maintaining a good long-term visual acuity (VA) ≤ 0.2 logMAR (≥ 0.6 decimal) following PT for parapapillary choroidal melanoma

Negative predictive factors for VA ≤ 0.2 logMAR (≥ 0.6 decimal) following PT for parapapillary choroidal melanoma				
	Univariate	OR	95% CI	Multivariate
**Baseline characteristics**				
Patient factors				
Age at PT	**0.006**	1.03	1.01–1.05	**0.04**
Diabetes	0.69	–	–	–
Arterial hypertension	0.61	–	–	–
Anticoagulant therapy	0.36	–	–	–
Ocular factors				
VA before PT (logMAR)	** < 0.001**	81.5	18.8–448.9	0.10
Foveal detachment	**0.002**	2.57	1.5–4.7	0.27
Tumor factors				
LTD	0.07	–	–	–
Tumor height	**0.005**	1.54	1.2–2.1	0.20
Tumor abutting OD	**0.02**	1.96	1.12–3.49	0.85
Temporal tumor location	**0.001**	2.86	1.6–5.4	0.91
Distance from the fovea				
≤ 0.5 mm	**< 0.001**	9.41	4.1–23.7	**0.03**
**PT parameters**				
% of OD surface irradiated at				
50% of dose (30 GCE)	**0.004**	1.02	1.00–1.02	**0.02**
ON length (mm) irradiated at				
50% of dose (30 GCE)	**0.002**	1.29	1.1–1.5	0.88
% of macular surface irradiated at				
50% of dose (30 CGE)	** < 0.001**	1.03	1.02–1.04	** < 0.001**

*CGE* Cobalt gray equivalent*, GVO* good visual outcome*, LTD* largest tumor diameter*, OD* optic disc*, ON* optic nerve*, OR* odds ratio*, PT* proton therapy*, PVO* poor visual outcome

The overall mean follow-up was 120.8 ± 48.8 months (range 54.0–295.0), with no significant statistical difference between the two groups (*P* = 0.053). The GVO group was on average five years younger (Table 2), and older age at the time of PT was identified as an independent risk factor (*P* = 0.04) for poor long-term visual outcome (Table 5). None of the recorded general health data (diabetes, arterial hypertension, anticoagulant therapy) differed statistically between groups. Prior to PT, patients of the GVO group had a better median BCVA (logMAR 0.0; range −0.2 to 0.7) than those of the PVO group (logMAR 0.3; range −0.2 to 2.3) (Table 2), but initial logMAR VA did not show to be a risk factor for poor long-term VA in the multivariate analysis (*P* = 0.10; Table 5). The fovea was also found to be more often detached in the PVO group (54% vs. 31% in the GVO group, Table 2), but foveal detachment could as such not be identified as an independent risk factor (Table 5). Mean ocular axial length did not differ between the groups (Table 2).

The LTD (overall median = 12.0; range 5.0–17.9 mm) did not differ between the GVO and PVO groups (Table 2), but the median tumor was 0.3 mm thicker in the PVO group (3.4 vs. 3.1 mm, *P* = 0.004; Table 2). However, this difference was no negative predictive factor on multivariate analysis (Table 5). The location of the posterior tumor border was strongly correlated with long-term visual outcome. That is, whether the tumor abutted the optic disc (GVO vs. PVO: 34% vs. 52%; *P* < 0.001), or was positioned temporally to the fovea (25% vs. 49%; *P* < 0.001), or at a closer distance to the fovea (*P* < 0.001), increased significantly the probability of a poor long-term visual outcome (Table 2).

**Fig. 2:**
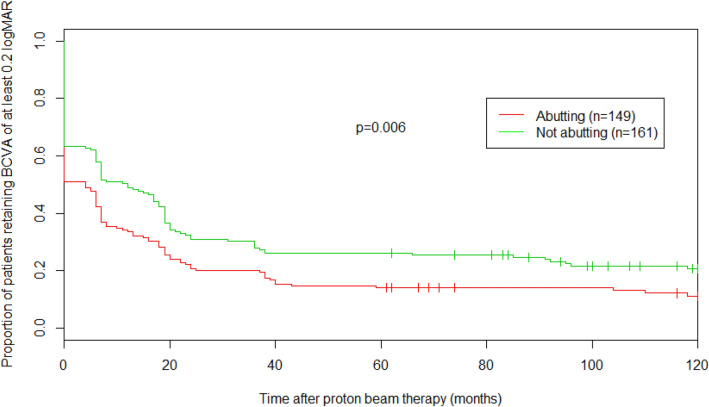
Kaplan–Meier survival curve (total No. of patients = 310), representing proportion of patients retaining good vision over time, according to the position of their posterior tumor border relative to the optic disc. *BCVA* best corrected visual acuity*, VA* visual acuity

**Fig. 3:**
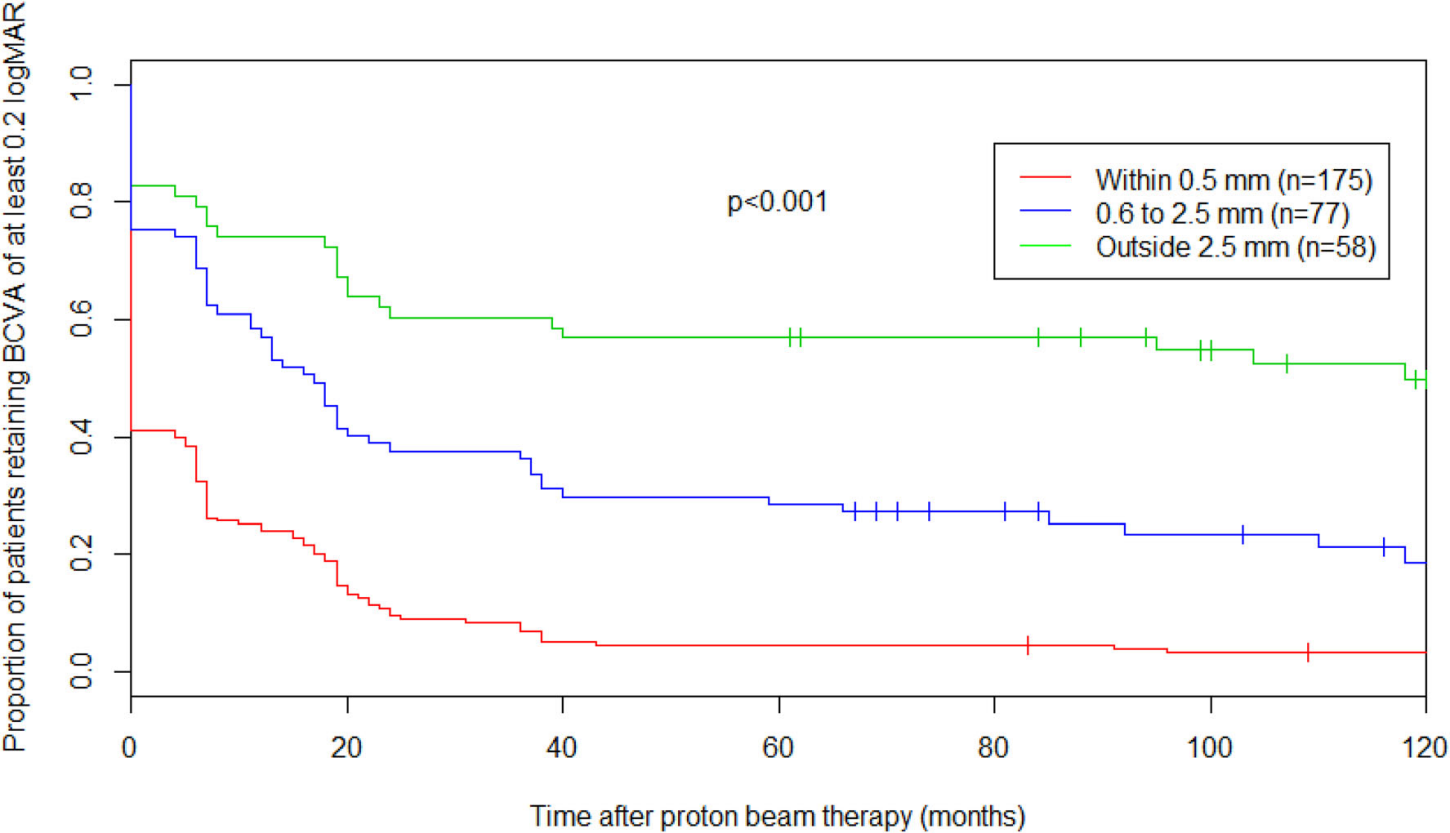
Kaplan–Meier survival curve (total No. of patients = 310), representing proportion of patients retaining good vision over time, according to the distance between their posterior tumor border and the fovea. *BCVA* best corrected visual acuity*, VA* visual acuity

In Fig. 2, the impact of the posterior tumor border location relative to the optic disc is shown via a Kaplan–Meier curve comparing the proportion of patients retaining good vision over time as a function of whether the tumor abutted the optic disc or not. Five years after PT, 14% (CI: 9–21%) of patients with tumors abutting the optic disc maintained a good vision compared to 26% (CI: 20–34%) of patients whose tumor did not touch the optic disc (*P* = 0.006). Figure 3 similarly emphasizes the importance of the posterior tumor border location with regard to the fovea, comparing the proportion of patients retaining good vision over time according to the distance between the tumor and the fovea (≤ 0.5 mm, 0.6–2.5 mm, > 2.5 mm). Five years after PT, only 5% (CI: 2–9%) of patients with tumors within 0.5 mm of the fovea maintained a good vision compared to 29% (CI: 20–41%) and 57% (CI: 46–71%) of patients whose tumor was within 0.6 and 2.5 mm or at more than 2.5 mm from the fovea, respectively (*P* < 0.001). Interestingly, on multivariate analysis, only the tumor location relative to the fovea remained significant with a distance of less than 0.6 mm from the fovea resulting as an independent negative predictor for maintaining good long-term VA (*P* = 0.03, Table 5).

The additional role of radiation-related parameters on long-term vision is shown in Tables 3, 4 and 5, where we show data of the GVO and PVO group divided according to the location of the posterior tumor border, i.e., abutting the disc or not (Table 3), and its distance to the fovea (Table 4). For similar tumor locations, GVO patients had received less irradiation to the optic disc, the optic nerve (Table 3) or the macula (Table 4). While the multivariate analysis identified both the % of irradiation delivered to the optic disc and to the macular surface as two independent negative predictors for maintaining long-term vision, it also highlighted the relative importance of irradiation delivered to the macula (*P* < 0.001), rather than the optic disc (*P* = 0.02), the former being identified as the strongest negative predictive factor for good long-term VA following PT (Table 5). The decade in which PT had been administered did not influence long-term vision in the patients of this study (*P* = 0.66). In line with previous findings, ocular follow-up data (Table 2) revealed radiation-induced maculopathy (GVO vs. PVO: 25% vs. 67%; *P* < 0.001) and optic neuropathy (28% vs. 47%; *P* = 0.007), as well as optic atrophy to have developed significantly more often in the PVO group.

## Discussion

This comparative study focuses on good VA outcomes following hypo-fractionated PT for small- and certain medium-sized pcM and identifies parameters correlated with long-term visual outcomes, varying widely in these patients with apparently similar tumors. We found that of 310 patients successfully treated between 1984 and 2005, 21% maintained a VA of ≤ 0.2 logMAR (≥ 0.6 decimal) and 43% a VA of ≤ 1.0 logMAR (≥ 0.1 decimal), for at least 5 years following PT. Independent negative predictive factors were older age at treatment, closer distance of the tumor to the fovea and a larger optic disc and macular surface area receiving at least 30CGE, the latter being the most significant risk factor for maintaining long-term VA.

A quantitative comparison between our long-term visual outcomes and those of the other PT centers is challenging, due to differences in the definition of pcM, treatment parameters, as well as study design, with other inclusion criteria. Furthermore, the definition of ‘Parapapillary’ choroidal melanoma differs between research groups. PcM is being defined as located at a maximum distance from the disc of 1 DD (~ 1.5 mm) by the Boston group [4], 0.5 mm by the Berlin group [5], 2 DD (~ 3 mm) by the Nice group [6] and 2.5 mm in the current study, corresponding to the maximal safety margins applied at the PSI. The Boston group has published most on the subject [2, 4, 14], but delivers a total radiation dose of 70CGE in 5 fractions instead of ± 60CGE in 4 fractions, used in the European centers.

Since these research groups [4–6] already reported on the global outcomes of pcM treated with PT, including survival, local tumor control, eye retention probability, visual outcomes and associated ocular complications, we opted for an alternative, comparative approach, trying to understand why certain PT-treated pcM patients maintained a surprisingly better long-term visual outcome than others. In consequence, our VA cut-off, separating a ‘GVO’ from a ‘PVO’ was stricter, i.e., ≤ 0.2 logMAR (≥ 0.6 decimal), instead of the usual ≤ 1.0 logMAR (≥ 0.1 decimal), the lower limit for ‘legal’ vision. This focus on excellent long-term VA also limited the upper cut-off value for tumor thickness to 5.2 mm, determined by the height of our thickest pcM case still maintaining ≤ 0.2 logMAR (≥ 0.6 decimal). Together with excluding T4 tumors with an LTD of more than 17.9 mm, we avoided confounding factors of ‘big tumor’ complications, such as retinal detachment and toxic tumor syndrome, in our statistical analysis. In consequence, our long-term VA outcomes should be interpreted as the results of a subgroup analysis, not as absolute, global results of PT for all our pcM patients. The fact that we had to exclude 295 of 609 patients due to missing follow-up data underscores the importance of this awareness.

Despite these limitations, our study highlights some important trends. First, maintaining a useful long-term vision following PT of a small- and even medium-sized pcM is not the exception. Even assuming a ‘worst case’ scenario, i.e., all 299 excluded patients being legally blind in the treated eye, then still 11% (64/609) would have maintained a VA of ≤ 0.2 logMAR (≥ 0.6 decimal) and 22% (64 + 70/609) a VA of ≤ 1.0 logMAR (≥ 0.1 decimal) (Table 1), which remain encouraging ‘worst case’ outcomes. We speculate that this proportion is improving, as the patients in this study were treated before the anti-VEGF era.

Second, patients with nasally located pcM have more chances of keeping good vision, the fovea apparently being relatively more radio-sensitive than the optic disc. In accordance with previous findings [4, 6], the threshold above which radiation-induced damages become substantial appears to be around 30 CGE. The reasons for these damages are likely related to the posterior choroidal vascular ultrastructure, as the disc benefits from a larger number of posterior ciliary arteries, usually four, assuring its vascular supply and offering a potential source of collateral compensating vessels, while the fovea is normally provided for by only two [15]. That is, future studies on intravitreal therapy for radiation-induced maculopathy or papillopathy ideally should match controls for location of the posterior tumor border with regard to both the fovea and optic disc to avoid biases.

Some observations of our study need to be considered with more caution, e.g., that younger age seems to be a protective factor for maintaining vision. Although Thariat et al. found the same correlation [6], Lane et al. came to the opposite conclusion [4], as did Matet et al. in a recent paper on vascular alterations in radiation induced maculopathy [16]. Differences in treatment parameters, selection criteria, or trend inversion over a longer follow-up period could be possible explanations for these opposing findings. The same applies to the fact that diabetes was not found to be a negative predictive factor in our study, contrary to the Boston and Nice series [4, 6]. Also, a worse initial VA and the irradiated length of the optic nerve were found to be significant negative predictors in other studies [6], but not in ours. A recent retrospective analysis of 1129 patients found the irradiated length of the optic nerve and the dose to the optic disc being risk factors for the development of radiation-induced optic neuropathy [17].

In conclusion, out of 310 successfully treated small- and even medium-sized pcM patients between 1984 and 2005, one in five maintained a VA ≤ 0.2 logMAR (≥ 0.6 decimal) for at least five years following PT. Independent negative predictors for maintaining a useful long-term vision were older age, tumor proximity to the fovea, and the volume of the optic disc and macula receiving at least 30 CGE, the latter being the most significant risk factor.
